# Characterization of telomere variant repeats using long reads enables allele-specific telomere length estimation

**DOI:** 10.1186/s12859-024-05807-5

**Published:** 2024-05-17

**Authors:** Zachary Stephens, Jean-Pierre Kocher

**Affiliations:** Quantitative Health Sciences, Mayo Clinic, Rochester, MN USA

**Keywords:** Telomeres, Telomere length, Long reads

## Abstract

Telomeres are regions of repetitive DNA at the ends of linear chromosomes which protect chromosome ends from degradation. Telomere lengths have been extensively studied in the context of aging and disease, though most studies use average telomere lengths which are of limited utility. We present a method for identifying all 92 telomere alleles from long read sequencing data. Individual telomeres are identified using variant repeats proximal to telomere regions, which are unique across alleles. This high-throughput and high-resolution characterization of telomeres could be foundational to future studies investigating the roles of specific telomeres in aging and disease.

**Supplementary Information:**

The online version contains supplementary material available at 10.1186/s12859-024-05807-5.

## Background

Telomeres are regions of repetitive DNA at the ends of linear chromosomes which are part of a biological system for protecting chromosome ends from degradation. Human telomeric DNA is comprised of (TTAGGG)n repeats, referred to as ’canonical repeats,’ which shorten with cell division in normal somatic cells. Telomeres can shorten to a point where they no longer protect chromosome ends, initiating cellular senescence, a state where cells do not further divide but retain biological function [1, 2].

**Fig. 1:**
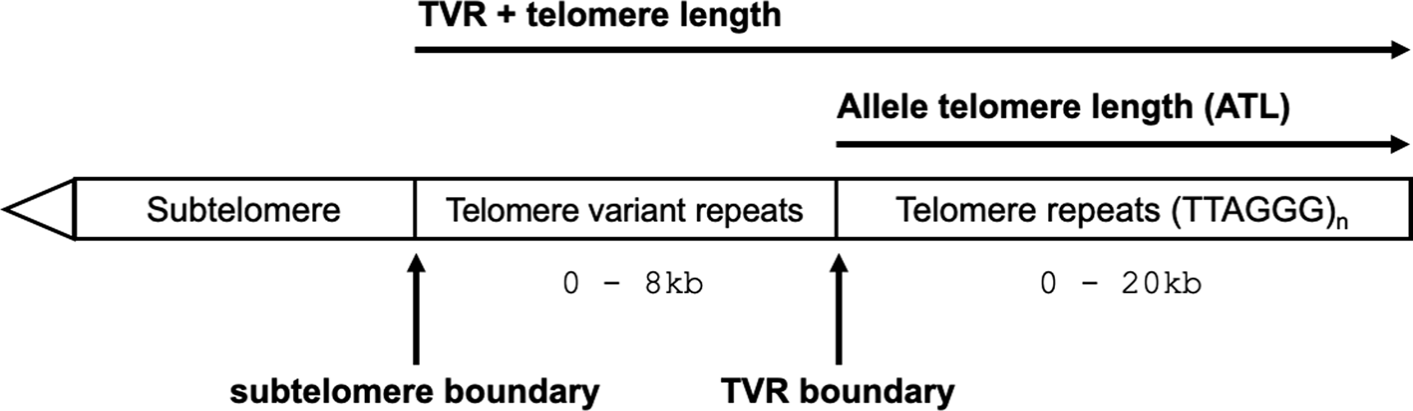
Organization of subtelomere, TVR and telomere regions at a chromosome end

The genomic regions adjacent to telomeres include telomere variant repeat (TVR) and subtelomere regions. In human, subtelomeres are informally defined as the most distal 500kb of each chromosome arm, and are repeat-rich and highly variable across populations [3, 4]. TVR regions are found between telomeres and subtelomeres (Fig. 1) and are composed of canonical repeats interspersed with blocks of ’variant repeats.’ The most frequent variant repeats are canonical TTAGGG sequences modified by a single base substitution (e.g. TCAGGG) or insertion / deletion (e.g. TTAAGGG). Early analysis of TVR regions identified the most common variant repeats [5], and subsequent studies reported a large diversity of repeat patterns both within individuals and across distantly related samples [6–8]. TVR regions are described as not having telomeric function due to the reduced binding affinity of variant repeats to shelterin genes TRF1 and TRF2 [9–11]. From targeted studies of telomeres 12q and Xp/Yp, it was found that the distributions of variant repeats within human TVR regions are highly variable across chromosome arms, but also subject to Mendelian inheritance. Thus it is thought that TVR regions exhibit relatively high de novo mutation rates and are specific to individual chromosomes [12, 13].

While the size and sequence composition of TVR regions has been studied in detail at a limited number of chromosome arms, TVR regions have not been broadly characterized genome-wide due to the lack of high-throughput methods capable of analyzing telomere sequences of individual alleles. The variable size and composition of TVR regions potentially impacts analyses of telomeres themselves: E.g. if a TVR region does not have telomeric function but is considered to be part of a telomere, from the perspective of a telomere length (TL) measurement method, then the TLs reported by the method will be an overestimate proportional to the size of the TVR region [14].

Methods for measuring TLs are of great interest due to the extensive relationships established between telomeres and aging, disease, and behavioral health. Typically, studies involving TL use results from methods that estimate the average length of a sample’s telomeres across all chromosome arms. These average values have proven useful in establishing correlations but are of limited use in characterizing the underlying mechanisms relating telomeres to disease [15, 16]. The length of a sample’s shortest telomere is often more informative than their average TL [17, 18], and the lengths of telomeres on specific chromosome arms have been observed to correlate with disease risk [19–21]. Since methods for measuring TLs of individual chromosome arms are generally low throughput, low resolution, or labor intensive [14, 22], recent approaches using nanochannel arrays or long read sequencing have been developed to address these limitations [23–25]. Long read sequencing in particular has garnered significant interest for telomere analysis, as recent improvements in cost and throughput make it increasingly accessible to clinical laboratories.

Current long read sequencing platforms can generate high-quality reads $20-30$kb in length that are capable of spanning telomeres and their adjacent regions. Several methods have recently been developed for analyzing telomeres with this data: Reed et al. [26] demonstrated the viability of aligning long reads to telomere boundaries for estimating telomere lengths, and EdgeCase [24] further showed that hierarchically clustering reads based on their sequence can separate individual telomeres. Telogator [27] builds upon these ideas and leverages the telomere-to-telomere (T2T) reference genome to report telomere lengths at each chromosome arm. ChArmTelo [28] is a recently described method for analyzing telomeres at individual chromosomes using 10X linked-reads, however the software is not publicly available and the protocols for sequencing 10X linked-reads is no longer supported. A new method for selectively enriching telomere regions using ’telobaits’ prior to long read sequencing has shown potential in reducing the cost of large studies via sample multiplexing [25]. However, the 5-8kb DNA fragments generated by this approach are limited in their ability to span entire telomeres or to extend far enough into subtelomeres such that reads can be uniquely mapped to specific chromosome arms. Additionally, the associated bioinformatics workflow only reports average TL alongside a simplified representation of TVR sequence composition that is limited in its ability to represent the variety of TVR patterns found in human telomeres.

To facilitate high-resolution characterization of individual telomeres and their adjacent TVR regions, we developed Telogator2, a method for reporting allele-specific telomere length (ATL) and TVR sequences from long read sequencing data. Telogator2 significantly expands the functionality of our previously released Telogator, going beyond chromosome arm specificity to report TVR and telomere sequences for individual alleles. Telogator2 can identify distinct telomere alleles in the presence of sequencing errors and alignments where reads may be mapped (or multi-mapped) to chromosome arms different from where they originated.

## Results

### Telogator2 overview

**Fig. 2:**
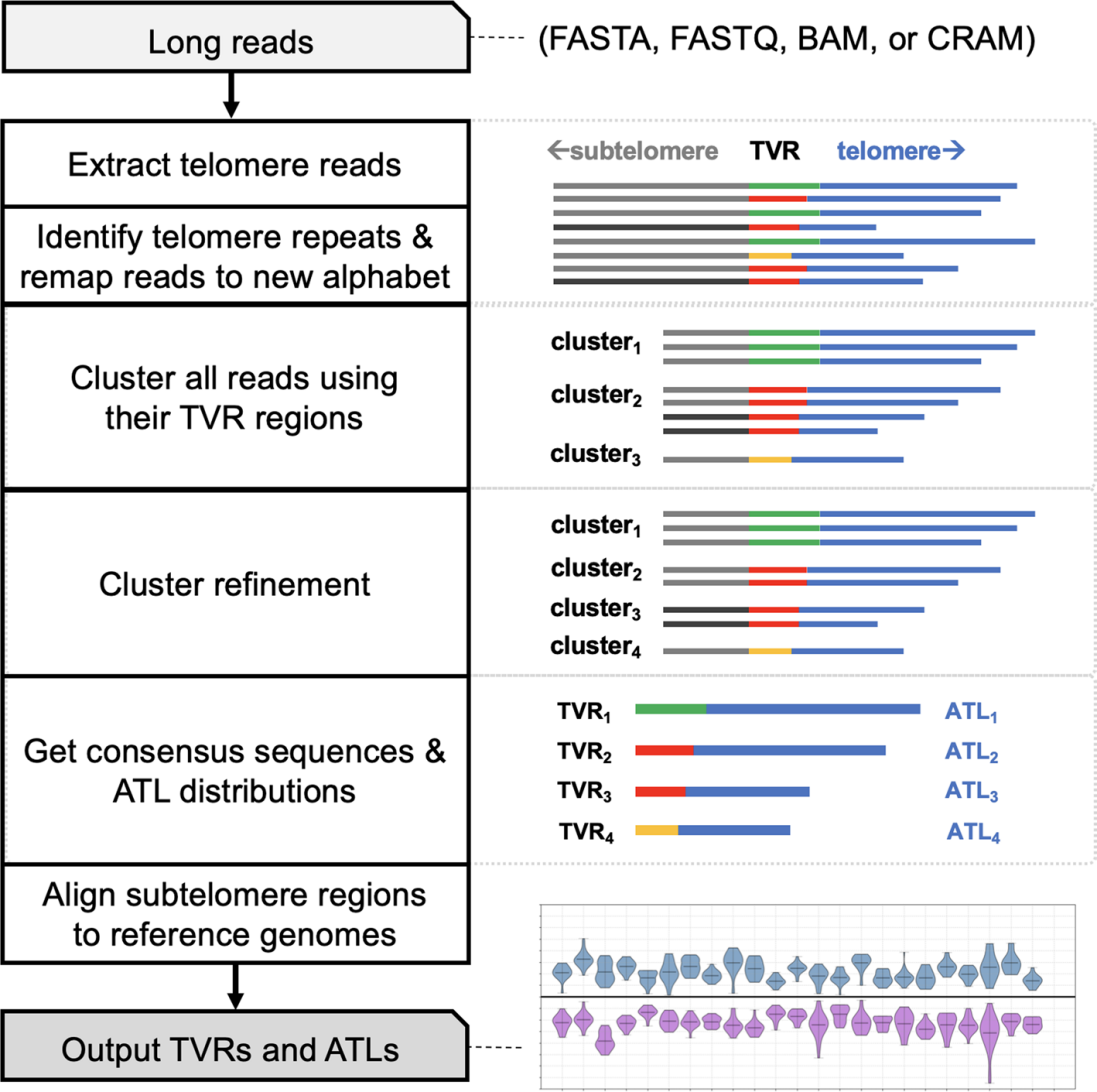
Overview of Telogator2. On the right are pictorial representations of telomere reads at each step

Telogator2 takes long reads as input, and begins by extracting a subset of reads containing a minimum number of canonical telomere repeats (10 copies by default). Telomere region boundaries are estimated based on the density of telomere repeats in a 100bp sliding window (described previously [27]), and reads that terminate in telomere sequence on one end and non-telomere sequence on the other are selected for further analysis. These reads are further processed to identify individual alleles based on their composition of telomere variant repeats. This is done by, i) querying reads for matches to a curated set of telomere repeats, ii) identifying TVR regions, and iii) clustering reads based on their TVR and subtelomere sequences (Fig. 2). After clustering, the subtelomere portions of each read are aligned to a customized reference genome containing subtelomere sequences from multiple telomere-to-telomere reference assemblies: T2T-CHM13 [29], as well as two diploid assemblies derived from Han Chinese individuals [30, 31]. Reads that can be aligned to a specific subtelomere are referred to as ’anchored’ telomeres. Finally, an output report is produced containing the lengths and sequence compositions of all anchored telomere alleles. Plots showing each allele are also produced, with TVR and telomere regions colorized based on their composition of variant repeats.

Telogator2 requires reads with low sequencing error rates ($<5\%$), and with lengths long enough to span a sample’s longest telomeres (plus at least 1kb adjacent subtelomere sequence for chromosome arm assignment). Telogator2 was primarily tested with $20-30$kb long reads from PacBio Sequel IIe, PacBio Revio and Oxford Nanopore (ONT) PromethION 2 platforms. Further considerations on input sequencing data are described in the Discussion.

### Validation of telomere alleles in CHM13

**Fig. 3:**
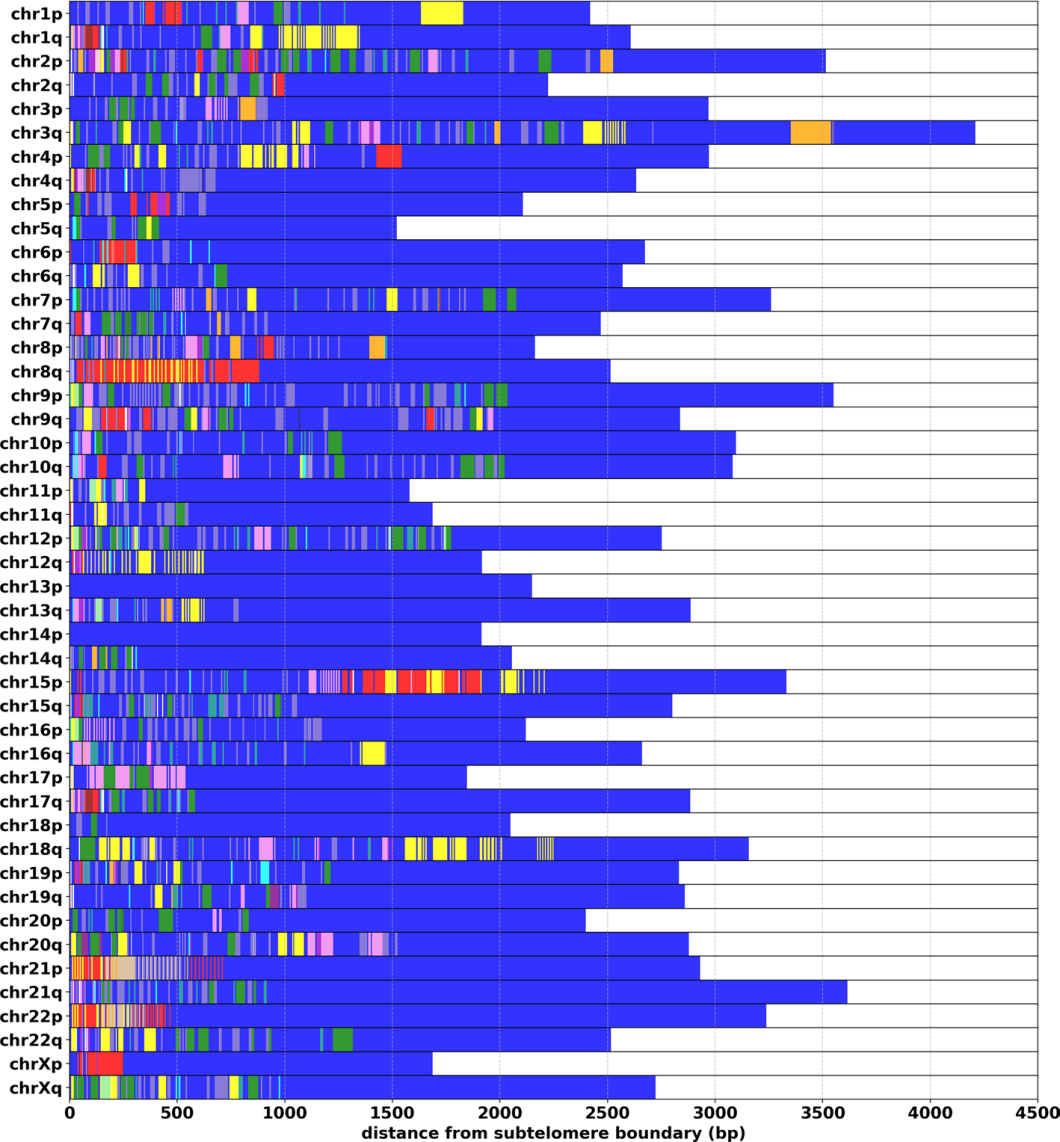
All telomere alleles in CHM13. Each row is the consensus TVR + telomere region for each chromosome arm. Blue regions are canonical TTAGGG repeats, and other colors are different variant repeats (full table of colors is provided in the Methods section). The TLs shown represent the 75th-percentile TL at each arm

As an initial validation, we analyzed PacBio HiFi reads of the CHM13 haploid cell line and compared the telomere alleles identified by Telogator2 to that of the T2T reference genome. From this data, Telogator2 identifies all 46 alleles, with one at each chromosome end (Fig. 3). 45/46 alleles are anchored at the expected reference coordinates at their respective arm’s subtelomere-telomere boundary. The one exception is 18p, where telomere reads were instead mapped $\sim 100$kb inside the subtelomere. Interestingly, the telomere for 18p was also absent from the initial de novo assemblies used to construct the T2T reference, and had to be resolved manually [29].

### Nearly all alleles have unique TVR regions

To examine TVR regions in a larger set of samples, we downloaded HiFi long reads from 46 samples that were sequenced as part of the Human Pangenome Reference Project [32], which selected healthy individuals from a variety of demographics. Each sample had 30-40x coverage in subtelomere regions, with the exception of HG02572 which was discarded as an outlier due to its lower coverage, leaving 45 samples in total. Processing these samples with Telogator2, we identified an average of 89 (std=3) unique alleles per sample, very close to the expected total number of alleles. This suggests that, assuming sufficient coverage and read lengths, and outside of situations where parents are closely related, each of an individual’s 92 telomeres can be distinguished based on their unique TVR and subtelomere sequences.

### TVR region length and repeat composition

**Fig. 4:**
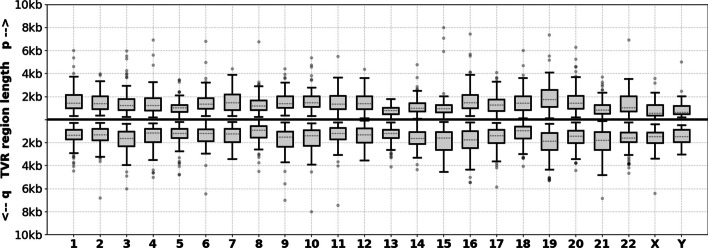
Distributions of TVR region lengths by chromosome arm for 45 human samples. The distributions on the top and bottom correspond to TVR region lengths at the p and q arms for each chromosome, respectively

We observe that, when averaged across samples, the lengths of TVR regions are very similar for most chromosome arms (Fig. 4). The exceptions to this are the TVR regions in the short arms of acrocentric chromosomes (particularly 13p, 14p and 15p) which on average are shorter than TVR regions from other arms. On average, the sequence composition of TVR regions is $62\%$ canonical TTAGGG repeats. The most frequent variant repeats: TGAGGG (G-type), TTGGGG (J-type) and TCAGGG (C-type), comprise $7\%$, $7\%$ and $4\%$ of TVR regions, respectively. Septamer repeats TTTAGGG and TTAGGGG collectively comprise $10\%$, and the remaining $10\%$ are rarer variations (full table provided in the Methods section).

While previous work has described TVR regions as being between 0-2kb [7, 33, 34], we observe that they can be as long as 8kb in some alleles (Fig. 4). We speculate that these larger TVR regions could potentially affect the accuracy of experimental methods for measuring the lengths of individual telomeres. For example, when using TRF [35] or STELA [36], it is common practice to subtract a constant corresponding to the distance between the telomere-subtelomere boundary and a reference point further into the subtelomere (an enzyme restriction site in the case of TRF, and subtelomere-specific primer sets for STELA). While this constant is typically in the range of 2-4kb [14], for alleles with very large TVR regions this may be insufficient and could result in overestimated ATLs.

### TVR region inheritance

To validate the sizes and sequence compositions of TVR regions reported by Telogator2, we processed long reads from the Han Chinese trio (HG005, HG006 and HG007) [37]. TVR regions are known to be inherited, thus the variant repeat patterns of a child should also be found in one of their parents with minimal variation. In total, we identified 91 unique alleles in child (HG005), 45 of which could be traced to the father (HG006), 45 traced to the mother (HG007), and a single allele containing very few variant repeats anchored to 22p where inheritance could not be determined. We speculate that the 92nd allele may have been missed either due to insufficient coverage from that particular allele, or that it may be highly similar to another allele such that the reads were clustered together.

**Fig. 5:**
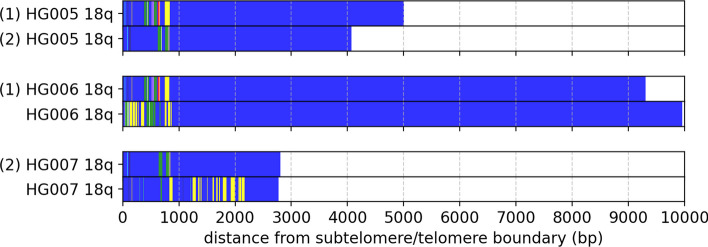
TVR and telomere regions of chromosome 18q for HG005 (son), HG006 (father) and HG007 (mother). Allele (1) is paternally inherited and allele (2) is maternally inherited

87 of the 90 inherited TVR regions appear to be virtually identical between parent and child (Example shown in Fig. 5). In a previous study of telomeres in trios, it was observed that the combined TVR + telomere regions in child alleles were closer in sequence similarity to their father than their mother [24]. However, when restricted solely to TVR regions we find no significant difference in the distances between HG005 / HG006 (average sequence similarity: $98.3\%$, std=$1.8\%$) and HG005 / HG007 (average sequence similarity: $98.0\%$, std=$2.0\%$). We describe the three alleles in HG005 that are were different than the corresponding allele in parent in the discussion section.

### TVR region uniqueness enables ATL estimation

For each set of reads from the same allele, a TL distribution can be estimated by simply reporting the length of the canonical repeats beyond the TVR boundary in each read. From these distributions a ’representative’ length for the allele can be chosen, e.g. by taking the mean or a percentile.

While the usage of whole genome sequencing for estimating average TL has been widely adopted and used in a large number of studies, the TL distributions for individual alleles are potentially more sensitive and could be affected by how the sample was prepared for sequencing. For example, fragmenting DNA to achieve a targeted size may introduce reads with incomplete telomere regions that have fewer canonical repeats than the allele from which it originated. To address this, several telomere capture protocols have recently been developed for preparing DNA for long read sequencing while ensuring that each molecule contains entire telomeres. An approach using ’telobaits’ [25] and PacBio sequencing has shown promise, however, the publicly available data from these preparations show limitations: Most notably, the average read lengths are comparatively short ($5-8$kb). Additionally, a majority of reads do not contain enough subtelomere sequence to be confidently assigned a unique chromosome arm. Another approach using ONT sequencing has recently been developed [38], but data generated from this protocol is not yet publicly available.

So while the ATLs presented in this work are all derived from whole genome sequencing, and thus should be interpreted as estimates, Telogator2 is readily applicable to reads sequenced following telomere capture using the methods listed above. As these experimental procedures improve and become more broadly accessible, we anticipate that Telogator2 will be a valuable method for analyzing the resultant data.

#### ATLs in CHM13

**Fig. 6:**
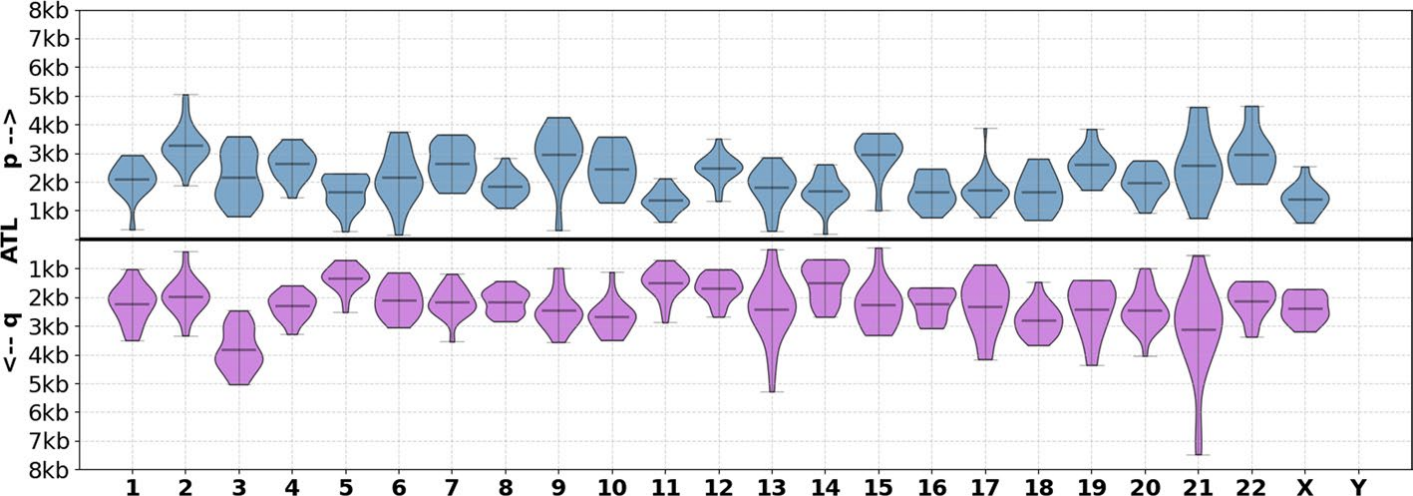
Distribution of ATLs at each chromosome arm of CHM13. The distributions on the top and bottom correspond to ATLs at the p and q arms for each chromosome, respectively

**Fig. 7:**
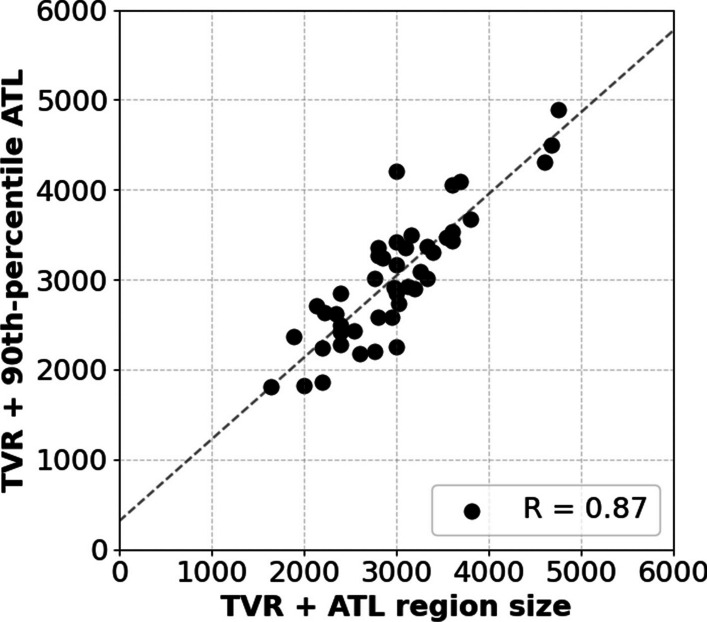
Correlation between CHM13 TVR + telomere lengths reported by Telogator2 (y axis) and the lengths of telomere region annotations in the T2T reference assembly (x axis). The 90th-percentile ATL was chosen as the representative length for each arm

Using the CHM13 telomere alleles identified earlier, we compared the ATL estimates reported by Telogator2 to the telomere sizes derived from T2T reference genome annotations. Because we are using the same whole genome long reads as were used in assembling the reference, we expect the ATL distributions found at each arm to correlate with the lengths of telomere regions in the final T2T reference assembly. We find that the ATLs reported by Telogator2 (Fig. 6) correlate well with the reference annotations ($R=0.87$), with an average difference of 288bp (std = 224bp) across all alleles (Fig. 7).

#### Variance in ATL across sequencing technologies

To assess the variability of ATLs across different sequencing technologies, we ran Telogator2 on long reads from HG002 (DNA acquired from Coriell), sequenced on both PacBio Revio and ONT PromethION 2 Solo platforms. The PacBio reads were combined from multiple runs for a total coverage of $\sim 75$x. The ONT reads were from a single run with a coverage of $\sim 20$x. In total we identified 90 unique alleles from the PacBio reads and 84 unique alleles from the ONT reads. We attribute this difference to coverage depth. All 84 alleles found from the ONT data could also be found in the PacBio data. For these 84 alleles we compared ATL distributions using multiple strategies for selecting a representative ATLs (Additional file 1). These strategies were tested in order to identify which method for selecting a representative ATL from a set of supporting reads is the most consistent across runs that are expected to have the same ATLs. We find that choosing the 75th percentile results in the highest correlation between HG002 ATLs between the PacBio and ONT datasets, with an average ATL difference of 588bp (std = 618bp).

Additionally, we compared the ATLs reported by Telogator2 on the 45 pangenome samples to average TL estimates produced by TelomereHunter [39] when applied to short reads from the same samples. For each sample, ATLs derived from the long reads were averaged together and compared against TelomereHunter’s ’tel_content’ output, yielding a correlation of $R=0.89$ (Additional file 2).

## Discussion

### TVR region uniqueness

A vast majority of telomere alleles in the samples we have analyzed have unique TVR regions that can be distinguished from each other. The non-unique TVRs fall into three categories:

- **False negatives**: TVR regions that have unique patterns, but are comprised of very few variant repeats such that they labeled by Telogator2 as ’blank’. These alleles are instead clustered using their subtelomere sequences.
- **No variant repeats**: Rare alleles where the canonical telomere repeats are directly adjacent to subtelomeres with no TVR region in between. We observe these alleles most frequently found on 22p and Xp.
- **Homozygous TVR regions**: TVR regions are inherited, thus it is possible that an individual could have two identical copies of a particular allele. E.g. If two parents are closely related, the telomeres of their offspring at some chromosome arms may be reported by Telogator2 as a single allele.

The default parameters in Telogator2 were tuned such that, in the presence of sequencing errors and other artifacts, that reads with the same TVR regions are clustered together in a vast majority of cases. Intermediate output plots are produced at each clustering step, so that individual thresholds can be adjusted by the user if necessary.

### De novo TVR variation

**Fig. 8:**
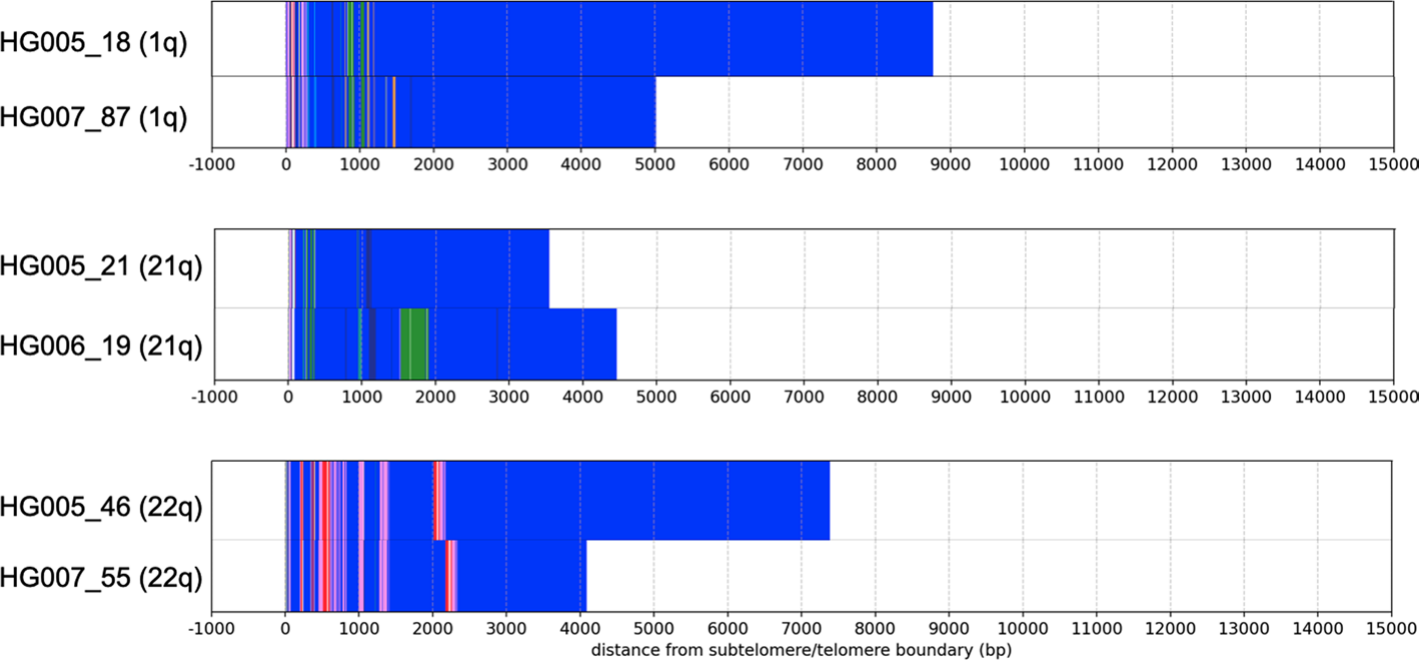
Inherited TVR + telomere regions in HG005 that have variation with respect to the corresponding allele in parent

A majority of the inherited TVR regions in the Han trio are extremely similar between parent and child (average sequence similarity $98.2\%$, std=$1.9\%$). However, three alleles had notable differences which we speculate could be attributable to de novo variation (Fig. 8):

- **HG005 1q**: Deletion of the most distal block of CCCTAG repeats with respect to maternal allele.
- **HG005 21q**: Deletion of the most distal block of CCCCAA repeats with respect to paternal allele.
- **HG005 22q**: Deletion within most distal block of canonical repeats within the TVR region, with respect to maternal allele.

All variation in these alleles involved deletions in the most distal portion of the TVR region (that is, near the variant repeats closest to the chromosome end). This is similar to observations made by Dubocanin et al. [40] that the most distal variant repeats can be ’somatically restructured’ and replaced with canonical repeats, possibly due to successive telomere erosion and elongation. However, in HG005 22q we observe that all blocks of variant repeats are intact and instead the most distal block of canonical repeats within the TVR region is shorter. Both observations suggest that the most distal positions of the TVR region are the most variable.

### Factors affecting ATL estimation from long reads

It is known that as cell populations proliferate their TLs become increasingly heterogeneous. When analyzing reads from a bulk sequencing experiment of normal somatic cells, the heterogeneity of TLs at a specific allele could represent different cells that have undergone different numbers of divisions. Thus, for such samples the ATLs are expected to have some variance attributable to this heterogeneity. Additionally, in a whole genome sequencing experiment where DNA is subjected to random fragmentation, there will be additional variance attributable to reads that only span a portion of a telomere.

**Fig. 9:**
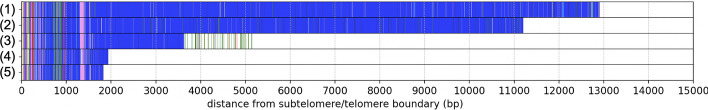
Reads from a 2p telomere allele from sample HG007, with reads (1) and (2) exhibiting abnormally long TLs

Cultured cell populations may also have additional factors affecting ATL distributions. All samples analyzed in this work originate from immortalized lymphoblastoid cell lines, the telomeres of which may not reflect the telomeres of the progenitor lymphocytes. We note that ATL distributions from these cell lines exhibit greater variance as compared to blood samples, and can have unexpectedly long telomeres at some alleles. For example, HG007 2p (Fig. 9) has reads with telomeres as long as 11kb, far greater than the sample’s average ATL of 2.3kb and not expected for a healthy individual that was 63 years old at the time of DNA acquisition.

Due to these factors, Telogator2 reports TL distributions for each allele. Users can either use these distributions directly, or specify a method for choosing a representative TL for each allele, such as max, median, or percentiles. Telogator2’s default is to report the 75th percentile ATL.

### Potential clinical applications of ATL

Allele-level characterization of telomeres provides a foundation for future studies to contrast samples with abnormal ATLs against baseline ATL distributions. For example, in the context of telomere biology disorders it is largely unknown whether low average TL, shortening of a specific telomere, or shortening of a group of telomeres is the most significant. A comprehensive characterization of ATL, beyond the limited number of arms that are usually studied in detail, using long reads and Telogator2 may be able to corroborate previous observations that the onset of cellular senescence is associated with the shortening a subset of telomeres beyond a critical length [41]. Similar analyses could be done in the context of cancer, where the eroded telomeres of individual arms could be studied for their associations with genomic instability or dysregulated gene expression.

### Limitations

#### Coverage depth

**Fig. 10:**
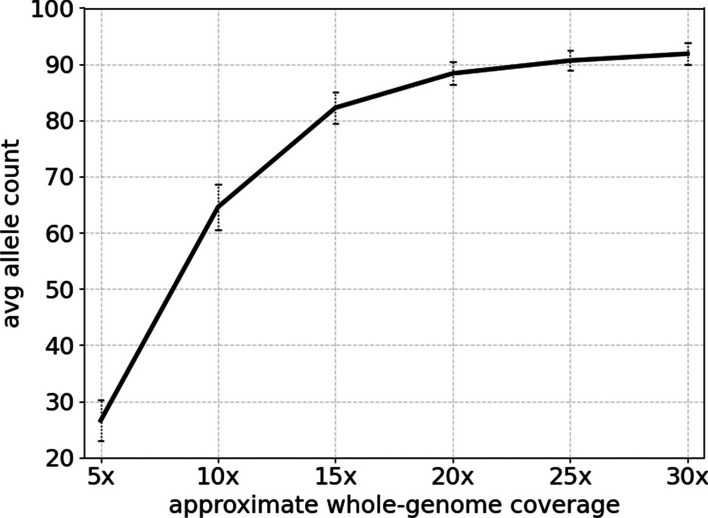
Average number of unique telomere alleles identified by Telogator2 when analyzing samples at different downsampled coverage depths

To assess the impact of coverage depth on Telogator2’s ability to identify telomere alleles, we selected 10 high coverage samples from the set of 45 human samples and conducted downsampling experiments. A typical run on a PacBio Sequel IIe will yield 10x whole genome coverage and $\sim 500$ anchored telomere reads. So we selected all samples from the pangenome set that had at least 1500 anchored telomere reads (corresponding to 30x whole genome coverage). We then generated reduced subsets of randomly selected reads corresponding to 5x, 10x, 15x, 20x, 25x, and 30x coverage. Each of these datasets was processed by Telogator2 with default parameters and a count of unique alleles was obtained. This procedure was repeated 10 times for each sample, for a total of $10\times 10\times 6 = 600$ experiments (Fig. 10). From this, we advise that a coverage depth of 30x is needed to have the best chance of identifying all alleles.

#### Subtelomere alignment uncertainty

**Fig. 11:**
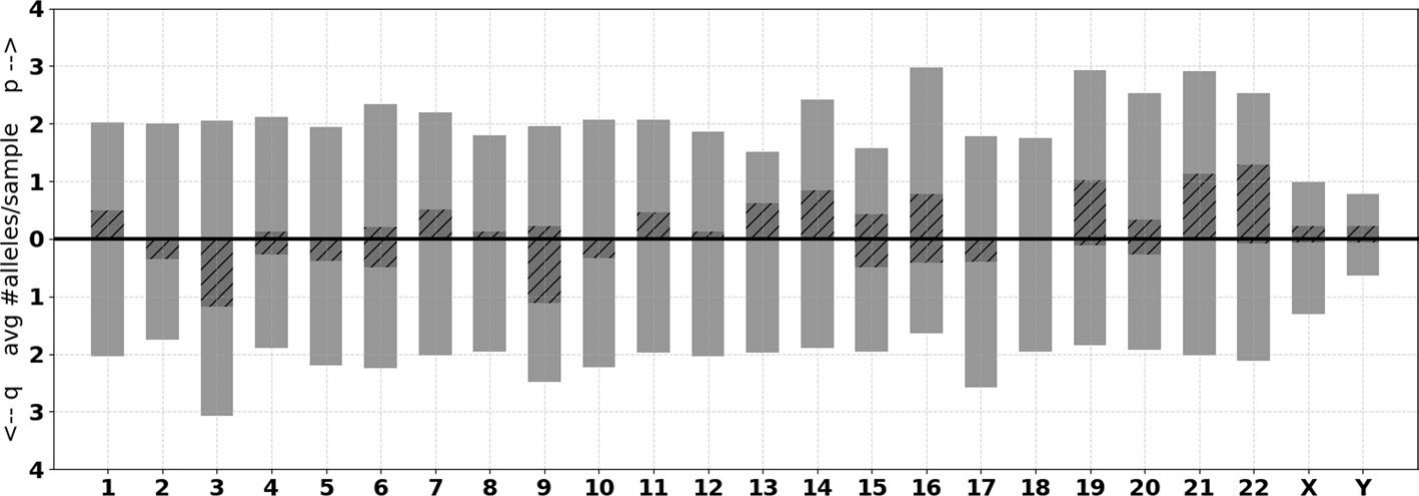
The average number of unique TVR regions aligned to each chromosome arm across 45 human samples. Shaded regions indicate the proportion of alleles that are multi-mapped and found at multiple arms

Due to the high sequence similarity of subtelomeres, it is expected that some telomere-spanning reads may be anchored to a subtelomere different from where they originated. By enumerating the average number of alleles anchored to each chromosome arm (Fig. 11), we observe that certain arms (most notably 3q, 16p, 19p and 21p) are assigned, on average, more than two alleles, indicating that alleles from other arms are being mapped there. Conversely, certain arms (such as 13p and 15p) are assigned less than two alleles, indicating that their subtelomeres are being mapped elsewhere.

A majority of arms are consistently found to have two alleles and minimal multi-mapping, particularly 1q, 2p, 3p, 4p, 5p, 7q, 8q, 11q, 12q, 13q, 14q, 17p, 18p, 18q, 21q and 22q. The stability of these arms may make them good candidates for validating experimental methods for characterizing individual telomeres. As additional high-quality telomere-to-telomere reference assemblies become available we will add them to the subtelomere reference set used by Telogator2, likely improving the uniformity of chromosome arm assignments.

#### Sequencing errors in telomere regions

While the sequencing error rates of long read technologies are consistently improving, with recent PacBio and ONT protocols achieving $<1\%$ error on average, we find that TVR and telomere regions generally exhibit higher error rates. In our analysis of PacBio reads we found that small sequencing errors in telomere regions are manageable with filtering and denoising strategies, but larger systematic artifacts may still pose challenges (example shown in Additional file 3). We estimate that these larger artifacts affect $\sim 5\%$ of telomere-spanning reads. Due to the low frequency of these artifacts they do not typically affect TVR consensus sequences. However, they could introduce a measurement bias in alleles that only supported by a few reads.

In our analysis of existing ONT datasets (e.g. whole genome HG002 reads available on SRA under project accession PRJNA744329) we found that the rate of sequencing errors in telomeres was prohibitively high, with systematic errors similar to those described by Tan et al. [42], and we were unable to use this data for analyzing individual telomeres. However, with recent updates to ONT basecalling software (github.com/nanoporetech/dorado/), we found that the quality of telomere regions has dramatically improved and that Telogator2 is readily applicable to recently sequenced data.

#### Samples with very long telomeres

In some use cases, a sample’s TLs may exceed the length of the reads, such as in the analysis of a cancer sample with elongated telomeres. Reads that are shorter than the length of a telomere will be unable to span the entire telomere and also be anchored in enough subtelomere sequence. While the $20-30$kb read lengths produced by commonly used PacBio and ONT protocols are sufficient for many studies, even longer reads will be needed to characterize samples with very long telomeres. For such studies, approaches such as TCA [43] or STAR [44] may be more appropriate than long read sequencing.

## Conclusions

We have demonstrated that Telogator2 can use long reads to provide a high-resolution characterization of telomeres. Using their adjacent TVR regions, telomere reads are clustered together into individual alleles from which allele-specific TL can then be estimated. We anticipate that this high-resolution characterization will enable future studies to further explore relationships between specific telomeres or sets of telomeres and their potential roles in senescence and telomere-associated diseases.

## Methods

### Telomere repeat set

Telogator2 uses a curated set of telomere repeats for identifying TVR regions (Table 1). These sequences were derived from analysis of PacBio HiFi long reads from multiple publicly available human samples, starting with the Han Chinese trio (acquired from the Sequence Read Archive under project accession PRJNA200694), which were chosen for their high coverage. We began by querying the reads for the canonical TTAGGG repeat and common variants such as TCAGGG (C-type), TGAGGG (G-type) and TTGGGG (J-type). Collectively, these four repeats comprised $\sim 90\%$ of all read sequence beyond the subtelomere boundary. The remaining regions that did not match any of these repeats were manually examined, and additional variant repeats were added to the set as they were found.

In the process of identifying variant repeats from this data, we observed an increased frequency of sequencing errors in telomere regions compared to reads aligned elsewhere in the genome. Specifically, we observed an increased frequency of insertion / deletion sequencing errors in homopolymer sequences, which is known to be a common sequencing artifact in long reads [45]. The J-type TTGGGG repeats are particularly prone to this error. To increase variant repeat matches in the presence of these errors additional patterns were added to the set representing T-type and J-type repeats modified by insertions and deletions.

### Identifying telomere regions

**Fig. 12:**
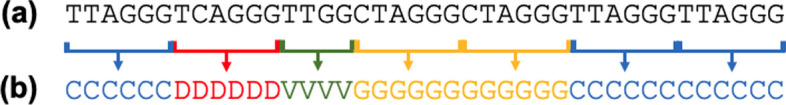
An example conversion from a nucleotide string **a** to a repeat symbol representation **b**

Telomere regions of each read are queried for exact matches to sequences from the telomere repeat set. Overlapping matches are prevented by searching for the longest repeats before searching for shorter repeats, while disallowing matches to a repeat if a larger repeat (that contains the smaller one) has already been found at that position. Partially overlapping matches are trimmed such that each position in the read is matched to a single telomere repeat from the variant repeat set. From these matches, each read is converted from a nucleotide string of [A,C,G,T] characters to a new string with symbols indicating which telomere variant repeat was found at that position (Fig. 12). The symbols corresponding to each repeat are shown in Table 1. This approach is similar to the ’telomere variant repeat codes’ used in visualizations by Baird et al. [13], and provides a representation of telomere sequences that simplifies downstream analysis via reducing repetitive DNA elements to contiguous blocks of identical symbols. To reduce the impact of sequencing errors leading to false positive matches, we prune singleton matches to canonical repeats modified by homopolymer insertions / deletions (e.g. TTAGGGG, TTAGG). For example, if an isolated TTAGGGG match is found, flanked on either side by normal canonical repeats, then it will be discarded and treated as if it were canonical. This is based on the observation that most sequencing errors in telomere regions are sparse and occur in isolation.

**Table 1:** Telomere repeats and their corresponding character mappings used by Telogator2. The repeat symbols are drawn from the amino acid alphabet so that external methods for sequence alignment can be used in subsequent steps

	Sequence	Symbol	Plot color
Canonical	TTAGGG	C	blue
C-type	TCAGGG	D	red
G-type	TGAGGG	E	yellow
J-type	TTGGGG	F	green
	CGAGGG	G	tan
	CTAGGG	H	orange
Common	CTGGGG	I	purple
variations	TAAGGG	K	cyan
	TCCGGG	L	pink
	TTCGGG	M	violet
	CTAGG	N	darkorange
Rare	TCGGG	P	darkviolet
variations	TGGGGG	Q	lightgreen
	TTAAGGG	R	darkcyan
	TTAGAGGG	S	gold
	TAGG^1^	T	darkblue
Errors	TTGG^2^	V	darkgreen
	GGGGGG	W	gray

^1^ Also includes TAGGG and TAGGGG

^2^ Also includes TGGG and TTGGG

#### TVR region clustering

**Fig. 13:**
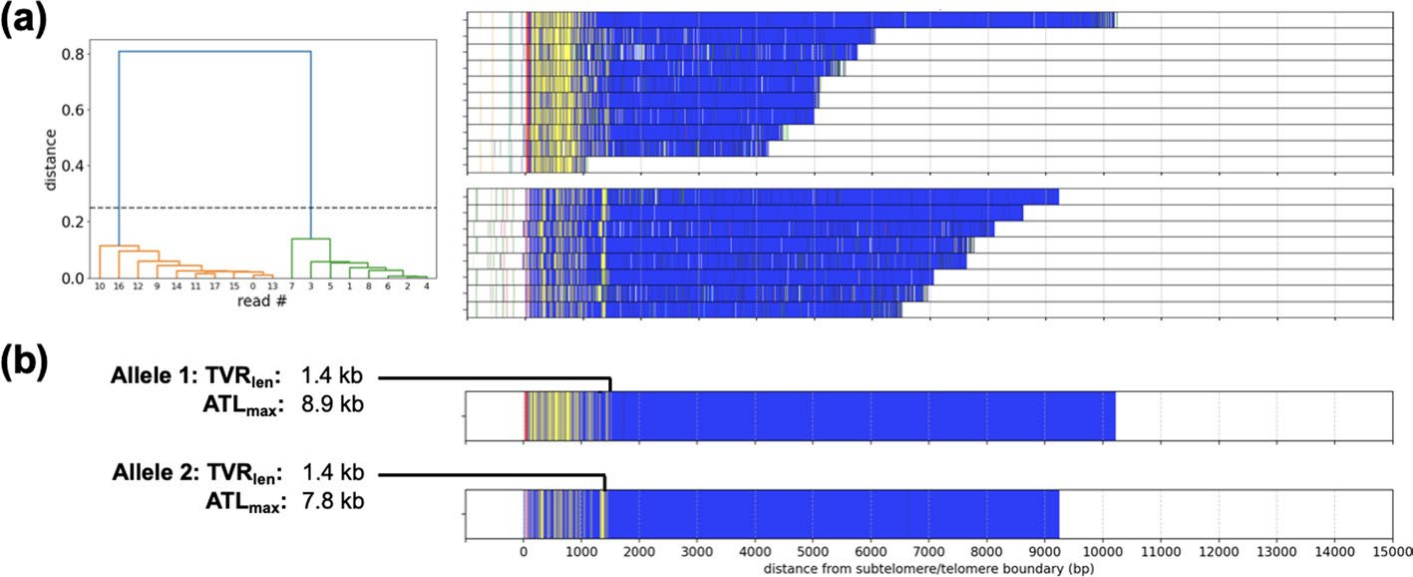
An example of TVR region clustering and boundary detection. (**a**) Hierarchical clustering of reads based on TVR patterns. (**b**) Estimation of TVR region boundary and ATL

TVR regions of all reads are then compared pairwise. Similarity scores are computed via pairwise alignment using Biopython [46] with a match score of +5 and mismatch / gap penalties of -4. These pairwise similarity scores are then converted to pairwise distances in the same manner as is done in progressive multiple sequence alignments [47]. Hierarchical clustering is then applied to the pairwise distances, and reads are assigned a cluster by cutting the resultant dendrogram (example shown in Fig. 13 (a)). Similar to previous Grigorev et al. [24], we found that the Ward variance minimization algorithm best separated the telomere alleles.

#### Cluster refinement

After forming initial clusters of reads, each cluster is processed individually in two steps: First, the TVR regions of reads are compared pairwise in the same manner as described above, but only to other reads in the same cluster, and using more aggressive clustering thresholds. This is done to separate distinct alleles that may have been clustered together in the first step, due their TVR regions sharing a common prefix or otherwise being similar enough to be grouped together. Second, the subtelomere portions of reads are compared to each other, again only to other reads in the same cluster. This is done to separate alleles where the TVR regions are highly similar, but the dissimilarity of the subtelomeres makes it clear that the supporting reads originate from a different allele. Overall, it is rare for clusters of reads to have highly similar telomeres and divergent subtelomeres, and we observed that this step only affects one or two clusters per sample, on average.

#### Consensus TVR sequences and ATL distributions

For each refined cluster, a consensus TVR sequence is obtained via multiple sequence alignment of its constituent reads using Muscle [48]. Muscle is run with a custom scoring matrix (Additional file 4) specifying the same match / mismatch penalties as were used for the pairwise alignments, with the exception that the matching score of canonical repeats is reduced to 0. This scoring matrix is designed to produce multiple alignments that prioritize matching variant repeats and reduce the reward for aligning canonical repeats in between the blocks of variant repeats. In our experience this produces consensus sequences that more closely resemble the variant repeat patterns in the individual reads. Additionally, this consensus process further mitigates the effect of sequencing errors in individual reads on the reported TVR sequences.

The TVR boundary for each allele is then determined from its consensus sequence. We define the boundary to be the position of the most distal variant repeat. In practice, we choose the TVR boundary to be the position at which $95\%$ of cumulative variant repeats have been observed (example shown in Fig. 13 (b)), due to sporadic variant repeats further into the telomere that are likely attributable to sequencing errors.

ATL distributions are reported for each cluster based on how far each supporting read extends beyond the TVR boundary. A representative’ ATL for each cluster can be chosen via a user-specified method, either the mean, median, max, or a percentile of an ATL distribution (default: mean).

#### Subtelomere anchoring

In order to assign each cluster a chromosome arm, the subtelomere portion of all supporting reads for all clusters are extracted and aligned to a collection of curated subtelomere reference sequences. By default, minimap2 [49] is used for read alignment, but winnowmap [50] or pbmm2 (https://github.com/PacificBiosciences/pbmm2) can be used instead via input options. The curated subtelomere reference contains the most distal 500kb of each chromosome arm of contigs from three recently released T2T references [29–31], and is extensible to include the subtelomeres of additional references as they become available.

In most cases, all subtelomere sections of reads from the same cluster will align to the same subtelomere reference sequence, making chromosome arm assignment straightforward. However, in approximately 5–10% of clusters, subtelomere reads will be mapped to multiple subtelomeres. In these cases, chromosome arm assignment will be ambiguous, and all candidate arms will be included in the final output. By default Telogator2 requires reads have at least 1kb of subtelomere sequence in order to be aligned. Long reads from whole genome sequencing will nearly always have sufficient subtelomere sequence, but this may not be the case for reads sequenced using telomere-capture strategies.

After chromosome arms have been assigned to each cluster, the TVR consensuses and ATL distributions are written to an output report, along with plots showing TVR and telomere regions colorized based on their composition of variant repeats.

### Sequencing data

Whole genome PacBio HiFi long reads for the CHM13 human haploid cell line were downloaded from the Sequence Read Archive (SRA) under project accession PRJNA530776. Whole genome HiFi reads for the Han Chinese trio (HG005, HG006, HG007) were downloaded from SRA under project accession PRJNA200694. Whole genome HiFi reads for 46 human samples (sequenced as part of the Human Pangenome Project) were downloaded from SRA under project accession PRJNA701308. Corresponding short read data for these 46 samples was downloaded from SRA at ftp.sra.ebi.ac.uk/vol1/run/ERR398/ in the form of BAM files (reads aligned to GRChg38). Telomere reads from HG002 are included in the Telogator2 repository. A full list of samples and their coverage is provided in Additional file 5.

### Supplementary Information

**Additional file 1:** Correlation of ATLs computed from PacBio and ONT datasets for HG002, using different methods for choosing a representative ATL from length distributions.

**Additional file 2:** Correlation of average ATL computed by Telogator2 from long reads vs. average TL computed by TelomereHunter from short reads.

**Additional file 3:** An example systematic sequencing artifact affecting telomere regions.

**Additional file 4:** Scoring matrix used by Telogator2 during sequence alignment of TVR regions.

**Additional file 5:** Table of all long read data analyzed in this study.

## Data Availability

Telogator2 is written in python and is available at www.github.com/zstephens/telogator2. Installation requirements and usage instructions can be found in the repository’s README.

## References

[1] Harley CB, Futcher AB, Greider CW (1990). Telomeres shorten during ageing of human fibroblasts. Nature.

[2] Shay JW, Wright WE (2005). Senescence and immortalization: role of telomeres and telomerase. Carcinogenesis.

[3] Riethman H, Ambrosini A, Castaneda C, Finklestein J, Hu X-L, Mudunuri U, Paul S, Wei J (2004). Mapping and initial analysis of human subtelomeric sequence assemblies. Genome Res.

[4] Mewborn S, Lese Martin C, Ledbetter D (2004). The dynamic nature and evolutionary history of subtelomeric and pericentromeric regions. Cytogenetic Genome Res.

[5] Allshire RC, Dempster M, Hastie ND (1989). Human telomeres contain at least three types of g-rich repeat distributed non-randomly. Nucleic Acids Res.

[6] Coleman J, Baird DM, Royle NJ (1999). The plasticity of human telomeres demonstrated by a hypervariable telomere repeat array that is located on some copies of 16p and 16q. Hum Mol Genet.

[7] Lee M, Teber ET, Holmes O, Nones K, Patch A-M, Dagg RA, Lau LMS, Lee JH, Napier CE, Arthur JW (2018). Telomere sequence content can be used to determine alt activity in tumours. Nucleic Acids Res.

[8] Bluhm A, Viceconte N, Li F, Rane G, Ritz S, Wang S, Levin M, Shi Y, Kappei D, Butter F (2019). Zbtb10 binds the telomeric variant repeat TTGGGG and interacts with trf2. Nucleic Acids Res.

[9] Nishikawa T, Okamura H, Nagadoi A, König P, Rhodes D, Nishimura Y (2001). Solution structure of a telomeric DNA complex of human TRF1. Structure.

[10] Hanaoka S, Nagadoi A, Nishimura Y. Comparison of dna-binding activities between htrf2 and htrfl with htrf2 mutants. Mod Magnet Resonance. 2006;743–751.

[11] Conomos D, Stutz MD, Hills M, Neumann AA, Bryan TM, Reddel RR, Pickett HA (2012). Variant repeats are interspersed throughout the telomeres and recruit nuclear receptors in alt cells. J Cell Biol.

[12] Baird DM, Jeffreys A, Royle N (1995). Mechanisms underlying telomere repeat turnover, revealed by hypervariable variant repeat distribution patterns in the human xp/yp telomere. EMBO J.

[13] Baird DM, Coleman J, Rosser ZH, Royle NJ (2000). High levels of sequence polymorphism and linkage disequilibrium at the telomere of 12q: implications for telomere biology and human evolution. Am J Hum Genetics.

[14] Aubert G, Hills M, Lansdorp PM (2012). Telomere length measurement-caveats and a critical assessment of the available technologies and tools. Mutat Res Fundamental Mol Mech Mutagenesis.

[15] Vera E, Blasco MA (2012). Beyond average: potential for measurement of short telomeres. Aging.

[16] Lansdorp PM (2022). Telomeres, aging, and cancer: the big picture. Blood J Am Soc Hematol.

[17] Hemann MT, Strong MA, Hao L-Y, Greider CW (2001). The shortest telomere, not average telomere length, is critical for cell viability and chromosome stability. Cell.

[18] Xu Z, Duc KD, Holcman D, Teixeira MT (2013). The length of the shortest telomere as the major determinant of the onset of replicative senescence. Genetics.

[19] Zheng Y-L, Loffredo CA, Shields PG, Selim SM (2009). Chromosome 9 arm-specific telomere length and breast cancer risk. Carcinogenesis.

[20] Xing J, Ajani JA, Chen M, Izzo J, Lin J, Chen Z, Gu J, Wu X (2009). Constitutive short telomere length of chromosome 17p and 12q but not 11q and 2p is associated with an increased risk for esophageal cancertelomere length and esophageal cancer risk. Cancer Prevent Res.

[21] Barkovskaya MS, Blinova E, Konyahina J, Leonova M, Nepomniashchikch V, Demina D, Kozhevnikov V, Kozlov V (2019). Telomere length distribution on individual chromosome arms in patients with bronchial asthma. Bulletin Siberian Med.

[22] Montpetit AJ, Alhareeri AA, Montpetit M, Starkweather AR, Elmore LW, Filler K, Mohanraj L, Burton CW, Menzies VS, Lyon DE (2014). Telomere length: a review of methods for measurement. Nurs Res.

[23] Young E, Pastor S, Rajagopalan R, McCaffrey J, Sibert J, Mak AC, Kwok P-Y, Riethman H, Xiao M (2017). High-throughput single-molecule mapping links subtelomeric variants and long-range haplotypes with specific telomeres. Nucleic Res.

[24] Grigorev K, Foox J, Bezdan D, Butler D, Luxton JJ, Reed J, McKenna MJ, Taylor L, George KA, Meydan C (2021). Haplotype diversity and sequence heterogeneity of human telomeres. Genome Res.

[25] Tham C-Y, Poon L, Yan T, Koh JYP, Ramlee MK, Teoh VSI, Zhang S, Cai Y, Hong Z, Lee GS (2023). High-throughput telomere length measurement at nucleotide resolution using the pacbio high fidelity sequencing platform. Nat Commun.

[26] Reed J, Kirkman LA, Kafsack BF, Mason CE, Deitsch KW. Telomere length dynamics in response to dna damage in malaria parasites. IScience. 2021 24(2). 10.1016/j.isci.2021.102082 PMC7887396 33644714

[27] Stephens Z, Ferrer A, Boardman L, Iyer RK, Kocher J-PA (2022). Telogator: a method for reporting chromosome-specific telomere lengths from long reads. Bioinformatics.

[28] Guo M, Songyang Z, Xiong Y. Charmtelo enables large-scale chromosome arm-level telomere analysis across human populations and in cancer patients. Small Methods. 2023;2300385. 10.1002/smtd.202300385 37526331

[29] Nurk S, Koren S, Rhie A, Rautiainen M, Bzikadze AV, Mikheenko A, Vollger MR, Altemose N, Uralsky L, Gershman A (2022). The complete sequence of a human genome. Science.

[30] He Y, Chu Y, Guo S, Hu J, Li R, Zheng Y, Ma X, Du Z, Zhao L, Yu W, et al. T2t-yao: a telomere-to-telomere assembled diploid reference genome for han chinese. Genom Proteom Bioinf. 2023. 10.1016/j.gpb.2023.08.001 PMC11082261 37595788

[31] Yang C, Zhou Y, Song Y, Wu D, Zeng Y, Nie L, Liu P, Zhang S, Chen G, Xu J (2023). The complete and fully-phased diploid genome of a male Han Chinese. Cell Res.

[32] Wang T, Antonacci-Fulton L, Howe K, Lawson HA, Lucas JK, Phillippy AM, Popejoy AB, Asri M, Carson C, Chaisson MJ (2022). The human pangenome project: a global resource to map genomic diversity. Nature.

[33] Capper R, Britt-Compton B, Tankimanova M, Rowson J, Letsolo B, Man S, Haughton M, Baird DM (2007). The nature of telomere fusion and a definition of the critical telomere length in human cells. Genes Develop.

[34] Déjardin J, Kingston RE (2009). Purification of proteins associated with specific genomic loci. Cell.

[35] Moyzis RK, Buckingham JM, Cram LS, Dani M, Deaven LL, Jones MD, Meyne J, Ratliff RL, Wu J-R (1988). A highly conserved repetitive DNA sequence,(TTAGGG) n, present at the telomeres of human chromosomes. Proceed Natl Academy Sci.

[36] Baird DM, Rowson J, Wynford-Thomas D, Kipling D (2003). Extensive allelic variation and ultrashort telomeres in senescent human cells. Nat Genet.

[37] Wang Y-C, Olson ND, Deikus G, Shah H, Wenger AM, Trow J, Xiao C, Sherry S, Salit ML, Zook JM (2019). High-coverage, long-read sequencing of Han Chinese trio reference samples. Sci Data.

[38] Schmidt TT, Tyer C, Rughani P, Haggblom C, Jones JR, Dai X, Frazer KA, Gage FH, Juul S, Hickey S, et al. High resolution long-read telomere sequencing reveals dynamic mechanisms in aging and cancer. bioRxiv. 2023;2023–11. 10.1038/s41467-024-48917-7 PMC11189484 38890299

[39] Feuerbach L, Sieverling L, Deeg KI, Ginsbach P, Hutter B, Buchhalter I, Northcott PA, Mughal SS, Chudasama P, Glimm H (2019). Telomerehunter-in silico estimation of telomere content and composition from cancer genomes. BMC Bioinf.

[40] Dubocanin D, Sedeno Cortes A, Ranchalis JE, Real T, Mallory B, Stergachis A. Single-molecule architecture and heterogeneity of human telomeric DNA and chromatin. bioRxiv. 2022;2022–05.

[41] Kaul Z, Cesare AJ, Huschtscha LI, Neumann AA, Reddel RR (2012). Five dysfunctional telomeres predict onset of senescence in human cells. EMBO Rep.

[42] Tan K-T, Slevin MK, Meyerson M, Li H (2022). Identifying and correcting repeat-calling errors in nanopore sequencing of telomeres. Genome Biol.

[43] Kahl VF, Allen JA, Nelson CB, Sobinoff AP, Lee M, Kilo T, Vasireddy RS, Pickett HA (2020). Telomere length measurement by molecular combing. Front Cell Develop Biol.

[44] Luo Y, Viswanathan R, Hande MP, Loh AHP, Cheow LF (2020). Massively parallel single-molecule telomere length measurement with digital real-time PCR. Sci Adv.

[45] Goodwin S, McPherson JD, McCombie WR (2016). Coming of age: ten years of next-generation sequencing technologies. Nat Rev Genet.

[46] Cock PJ, Antao T, Chang JT, Chapman BA, Cox CJ, Dalke A, Friedberg I, Hamelryck T, Kauff F, Wilczynski B (2009). Biopython: freely available python tools for computational molecular biology and bioinformatics. Bioinformatics.

[47] Feng D-F, Doolittle RF (1987). Progressive sequence alignment as a prerequisite to correct phylogenetic trees. J Mol Evol.

[48] Edgar RC (2004). Muscle: multiple sequence alignment with high accuracy and high throughput. Nucleic Acids Res.

[49] Li H (2018). Minimap2: pairwise alignment for nucleotide sequences. Bioinformatics.

[50] Jain C, Rhie A, Zhang H, Chu C, Walenz BP, Koren S, Phillippy AM. Weighted minimizer sampling improves long read mapping. Bioinformatics 2020;36(Supplement_1):111–118. 10.1093/bioinformatics/btaa435 PMC7355284 32657365

