# Supplementary material for: Characterization of telomere variant repeats using long reads enables allele-specific telomere length estimation
Source: BMC Bioinformatics. 2024 May 17;25:194. doi: 10.1186/s12859-024-05807-5 (PMC11100205; doi:10.1186/s12859-024-05807-5)
Supplement: Supplementary file 2 — Additional file 2: Correlation of average ATL computed by Telogator2 from long reads vs. average TL computed by TelomereHunter from short reads. [file 12859_2024_5807_MOESM2_ESM.pdf]

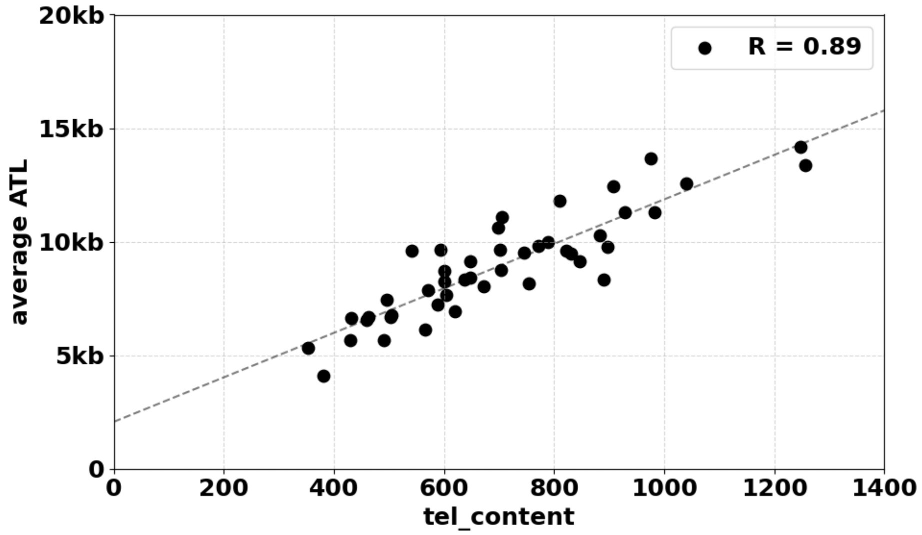

Correlation of average ATL computed by Telogator2 from long reads vs. tel_content computed by TelomereHunter from short reads. Short read BAMs (aligned to GRChg38) for the 45 pangenome samples were downloaded from SRA. These BAMs were processed by TelomereHunter, yielding a report of telomere length statistics for each sample. For each sample we then compared the "tel_content" metric produced by TelomereHunter to the average of the ATL values reported by our method. The average telomere length estimates from short reads are correlated ($R = 0.89$) with the average ATL observed in long reads, indicating that average telomere length measurements are largely unaffected by the variability of TVR regions. Average ATL in base pairs can be approximated from the tel_content value produced by TelomereHunter via: $ATL_{avg} \approx 10 \times tel\_content + 2000$.
