# Supplementary material for: Characterization of telomere variant repeats using long reads enables allele-specific telomere length estimation
Source: BMC Bioinformatics. 2024 May 17;25:194. doi: 10.1186/s12859-024-05807-5 (PMC11100205; doi:10.1186/s12859-024-05807-5)
Supplement: Supplementary file 3 — Additional file 3: An example systematic sequencing artifact affecting telomere regions. [file 12859_2024_5807_MOESM3_ESM.pdf]

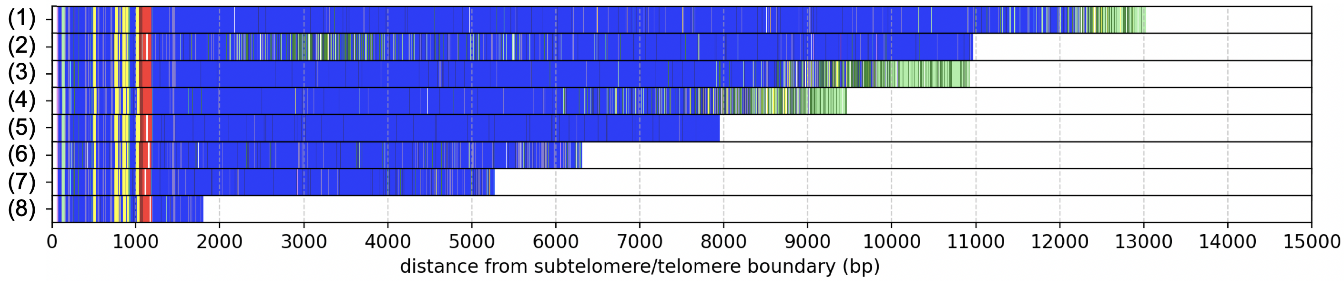

Reads from a 13q telomere allele from sample HG00423. Reads numbered (1), (2), (3) and (4) exhibit a systematic artifact found in certain PacBio HiFi reads where canonical TTAGGG repeats are miscalled as TTGGGG, TGGGGG, and similar variations.
