# Supplementary material for: Characterization of telomere variant repeats using long reads enables allele-specific telomere length estimation
Source: BMC Bioinformatics. 2024 May 17;25:194. doi: 10.1186/s12859-024-05807-5 (PMC11100205; doi:10.1186/s12859-024-05807-5)
Supplement: Supplementary file 4 — Additional file 4: Scoring matrix used by Telogator2 during sequence alignment of TVR regions. [file 12859_2024_5807_MOESM4_ESM.pdf]

	A	R	N	D	C	Q	E	G	H	I	L	K	M	F	P	S	T	W	Y	V	B	J	Z
A	2	0	0	0	1	0	0	0	0	0	0	0	0	0	0	0	0	0	0	0	0	0	0
R	0	5	-4	-4	-4	-4	-4	-4	-4	-4	-4	-4	-4	-4	-4	-4	-4	-4	-4	-4	-4	-4	-4
N	0	-4	5	-4	-4	-4	-4	-4	-4	-4	-4	-4	-4	-4	-4	-4	-4	-4	-4	-4	-4	-4	-4
D	0	-4	-4	5	-4	-4	-4	-4	-4	-4	-4	-4	-4	-4	-4	-4	-4	-4	-4	-4	-4	-4	-4
C	1	-4	-4	-4	0	-4	-4	-4	-4	-4	-4	-4	-4	-4	-4	-4	-4	-4	-4	-4	-4	-4	-4
Q	0	-4	-4	-4	-4	5	-4	-4	-4	-4	-4	-4	-4	-4	-4	-4	-4	-4	-4	-4	-4	-4	-4
E	0	-4	-4	-4	-4	-4	5	-4	-4	-4	-4	-4	-4	-4	-4	-4	-4	-4	-4	-4	-4	-4	-4
G	0	-4	-4	-4	-4	-4	-4	5	-4	-4	-4	-4	-4	-4	-4	-4	-4	-4	-4	-4	-4	-4	-4
H	0	-4	-4	-4	-4	-4	-4	-4	5	-4	-4	-4	-4	-4	-4	-4	-4	-4	-4	-4	-4	-4	-4
I	0	-4	-4	-4	-4	-4	-4	-4	-4	5	-4	-4	-4	-4	-4	-4	-4	-4	-4	-4	-4	-4	-4
L	0	-4	-4	-4	-4	-4	-4	-4	-4	-4	5	-4	-4	-4	-4	-4	-4	-4	-4	-4	-4	-4	-4
K	0	-4	-4	-4	-4	-4	-4	-4	-4	-4	-4	5	-4	-4	-4	-4	-4	-4	-4	-4	-4	-4	-4
M	0	-4	-4	-4	-4	-4	-4	-4	-4	-4	-4	-4	5	-4	-4	-4	-4	-4	-4	-4	-4	-4	-4
F	0	-4	-4	-4	-4	-4	-4	-4	-4	-4	-4	-4	-4	5	-4	-4	-4	-4	-4	-4	-4	-4	-4
P	0	-4	-4	-4	-4	-4	-4	-4	-4	-4	-4	-4	-4	-4	5	-4	-4	-4	-4	-4	-4	-4	-4
S	0	-4	-4	-4	-4	-4	-4	-4	-4	-4	-4	-4	-4	-4	-4	5	-4	-4	-4	-4	-4	-4	-4
T	0	-4	-4	-4	-4	-4	-4	-4	-4	-4	-4	-4	-4	-4	-4	-4	5	-4	-4	-4	-4	-4	-4
W	0	-4	-4	-4	-4	-4	-4	-4	-4	-4	-4	-4	-4	-4	-4	-4	-4	5	-4	-4	-4	-4	-4
Y	0	-4	-4	-4	-4	-4	-4	-4	-4	-4	-4	-4	-4	-4	-4	-4	-4	-4	5	-4	-4	-4	-4
V	0	-4	-4	-4	-4	-4	-4	-4	-4	-4	-4	-4	-4	-4	-4	-4	-4	-4	-4	5	-4	-4	-4
B	0	-4	-4	-4	-4	-4	-4	-4	-4	-4	-4	-4	-4	-4	-4	-4	-4	-4	-4	-4	5	-4	-4
J	0	-4	-4	-4	-4	-4	-4	-4	-4	-4	-4	-4	-4	-4	-4	-4	-4	-4	-4	-4	-4	5	-4
Z	0	-4	-4	-4	-4	-4	-4	-4	-4	-4	-4	-4	-4	-4	-4	-4	-4	-4	-4	-4	-4	-4	5

Scoring matrix used during multiple sequence alignment of TVR regions.
